# Pulmonary mucormycosis following autologous hematopoietic stem cell transplantation for rapidly progressive diffuse cutaneous systemic sclerosis: A case report

**DOI:** 10.1097/MD.0000000000021431

**Published:** 2020-07-31

**Authors:** Xavier Boumaza, Lucie Lelièvre, Sarah Guenounou, Cécile Borel, Anne Huynh, Guillaume Beziat, Karen Delavigne, Damien Guinault, Marie Garric, Marie Piel-Julian, Kim Paricaud, Guillaume Moulis, Leonardo Astudillo, Laurent Sailler, Dominique Farge, Grégory Pugnet

**Affiliations:** a Department of Internal Medicine; b Department of infectious and tropical diseases, Toulouse University Hospital; c Hematology Department; d Department of Internal Medicine, Institut Universitaire du Cancer de Toulouse - Oncopole; e Department of Nephrology and Organ Transplantation; f Clinical Investigation Center, Toulouse University Hospital; g UMR 1027 INSERM, University of Toulouse, Toulouse; h Unité de Médecine Interne: Maladies Auto-immunes et Pathologie Vasculaire (UF 04), Hôpital St-Louis, AP-HP, 1 Avenue Claude Vellefaux; i Centre de Référence des Maladies auto-immunes Systémiques Rares d’Ile-de-France; j EA 3518, Université Denis Diderot, Paris, France; k Department of Internal Medicine, McGill University, Montréal, Canada.

**Keywords:** autologous stem cells, mucormycosis, systemic sclerosis

## Abstract

**Rationale:**

The use of autologous hematopoietic stem cell transplantation (AHSCT) for autoimmune diseases has become the first indication for transplant in nonmalignant disease. Mucormycosis is a rare invasive infection with increasing incidence in patients treated with AHSCT. We report the first case of pulmonary mucormycosis following AHSCT for systemic sclerosis (SSc).

**Patient concerns:**

A 24-year-old woman with rapidly progressive diffuse cutaneous SSc presented with an acute respiratory distress syndrome 6 days after AHSCT.

**Diagnoses:**

The results of clinical and computed tomography scan were consistent with pulmonary mucormycosis and the diagnosis was confirmed by a positive Mucorales Polymerase Chain Reaction on a peripheral blood sample.

**Interventions and Outcomes:**

Early antifungal therapy by intravenous amphotericin B provided rapid improvement within 4 days and sustained recovery after 2 years of follow-up.

**Lessons:**

With the progressively increasing use of AHSCT and other stem cell therapy for treatment of severe SSc and other autoimmune diseases, the potential onset of rare post-transplant fungal infections, such as mucormycosis, requires careful patient monitoring and better awareness of early initiation of adequate therapy.

## 1 Introduction

Mucormycosis is a rare severe invasive fungal infection, caused by filamentous fungi of the class zygomycetes order Mucorales, primarily encountered in transplanted or diabetic patients.^[1–4]^ This severe disease occurs in highly immunosuppressed patients and is associated with a mortality rate ranging from 35% to 60%, depending on the associated comorbidities.^[5]^

Over the past 20 years the use of autologous hematopoietic stem cell transplantation (AHSCT) to treat severe rapidly progressive systemic sclerosis (SSc) has expanded progressively and this procedure is now recommended as a first-line option,^[6–8]^ on condition that the procedure is performed in an expert center, according to European Society for Blood and Marrow Transplantation guidelines.^[9–12]^ In such circumstances, careful patient pretransplant evaluation and close posttransplant follow-up are recommended,^[11,12]^ especially during the first 2 years after transplant while progressive immune reconstitution occurs. Mucormycosis has never been reported following AHSCT for autoimmune diseases or in SSc patients.

In this article we report on the first case of pulmonary mucormycosis after AHSCT for rapidly progressive diffuse cutaneous SSc.

## 2 Case

A 24-year-old woman diagnosed with SSc 2 years ago and who had no other past medical history, was initially treated with low-dose corticosteroids and mycophenolate mofetil for 9 months then with methotrexate for 6 months and 2 courses of rituximab. She was then referred for AHSCT due to a rapidly progressive disease with extensive skin fibrosis (modified Rodnan skin score 25/51) and pulmonary involvement [decreased diffusing capacity of the lung for carbon monoxide at 56% of the theoretical values on lung function tests and the presence of ground-glass infiltrate and diffuse bronchial wall thickening on chest computerized tomography (CT) scan]. Right heart catheterization with fluid overload, echocardiography, cardiac magnetic imaging, and myocardial scintigraphy were all normal.

Peripheral blood hematopoietic stem cells (PBSCs) were mobilized with intravenous (IV) cyclophosphamide (CPM) (total of 2 g/m^2^ administered over 2 consecutive days), and filgrastim (10 μ/kg/day for 7 days), allowing collection of 25 × 10^6^ CD34+ cells/kg in 2 cytaphereses.

Forty days later, a conditioning regimen with a total dose of 200 mg/kg IV CPM over 4 consecutive days with 2 L of 0.9% IV saline/day, plus IV rabbit antithymocyte globulins (2.5 mg/kg/day for 5 consecutive days) was administered followed by a reinjection of non-selected PBSC (day 0).

On day 0, the patient had leukopenia of less than 0.1 g/L and aplasia was sustained until day 12. She experienced her first febrile peak (39°C) the day after PBSC reinfusion while physical examination was otherwise normal and peripheral and port-a-cath blood cultures remained sterile. An empiric IV antibiotic therapy by piperacillin-tazobactam and amikacin was started. The patient remained afebrile until day 4 after PBSC reinjection, when she had a new onset of fever with a progressive increase in C-reactive protein level (278 mg/L). Repeated peripheral and port-a-cath blood cultures were sterile. The IV antibiotic therapy was switched to meropenem, ciprofloxacin, and vancomycin.

**Figure 1:**
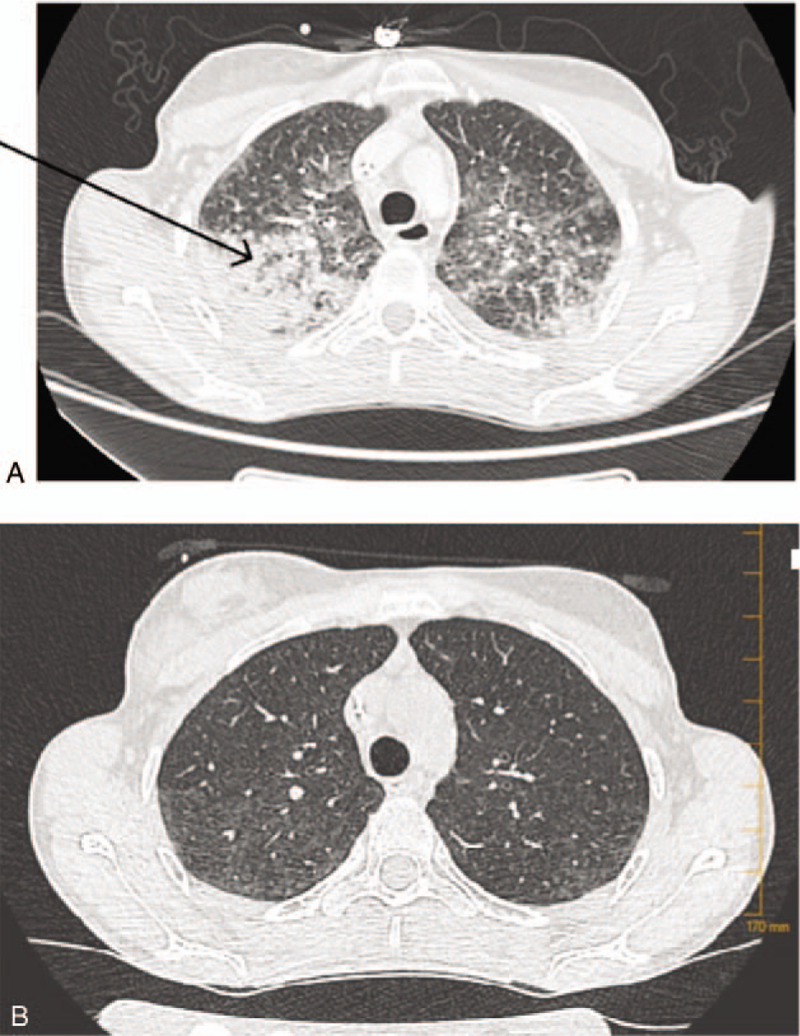
Pulmonary mucormycosis appearing as a mass with the halo sign on computerized tomography (CT) in 24-year-old woman after autologous hematopoietic stem cell transplantation for rapidly progressive diffuse cutaneous systemic sclerosis. A, CT scan on day 6, showing a bilateral parenchymatous condensation within the right upper lobe the reverse halo sign. B, CT scan on day 20 showing showed a nearly complete regression of bilateral parenchymatous condensation and disappearance of the reverse halo sign.

The patient remained febrile (39°C) and 2 days later developed a dry cough. The chest CT scan revealed massive bilateral pleural effusion, upper right lobe condensation, and less extensive upper left lobe condensation (Fig. 1).

On day 6, an acute respiratory distress syndrome with hypoxemia at 60 mm Hg required endotracheal intubation and mechanical ventilation. Acyclovir (10 mg/kg/dose/8 h IV) and amphotericin B (5 mg/kg/dose/24 h IV) were added. Repeated CT scan showed bilateral parenchymal condensation with a focal ground-glass opacity surrounded by a consolidation ring in the right upper lobe, consistent with the reverse halo sign (Fig. 1A).

Bronchoalveolar lavage (BAL) was performed and tests for cytomegalovirus, herpes simplex virus 1, herpes simplex virus 2, varicella zoster virus, adenovirus, other respiratory viruses, as well as for *Aspergillus* sp and mucormycoses (species tested for were *Mucor* sp., *Rhizopus* sp., *Lichtheimia* sp, and *Rhizomucor* sp.) by polymerase chain reaction (PCR) were all negative. Blood, Aspergillus antigenemia was negative, whereas *Mucor* sp. and/or *Rhizopus* sp PCR were positive.

The diagnosis of pulmonary mucormycosis was established on day 10 after AHSCT, and IV amphotericin B, increased to 7.5 mg/kg, with the addition of isavuconazole (200 mg/dose/8 h IV for 48 h and then 200 mg/24 h) were started. Antibiotics were discontinued and sulfamethoxazole-trimethoprim and valacyclovir were resumed at prophylactic doses. Rapid clinical improvement was observed, the patient was extubated on day 14 and oxygen therapy was stopped on day 17. Blood PCR for mucormycosis was negative on day 18.

The follow-up chest CT scan (on day 20) showed almost complete regression of bilateral alveolar parenchymal condensation and resolution of the reverse halo sign (Fig. 1B). Isavuconazole was stopped on day 21. Amphotericin B was discontinued on day 22 because of refractory hypokalemia due to tubular injury and loss of potassium. Maintenance therapy with posaconazole per os was administered from day 21 until day 28 (patient's decision) and the patient was discharged on day 22 after AHSCT.

After 1 year of follow-up, the patient is still in clinical and CT scan remission from mucormycosis.

## 3 Discussion

Invasive mucormycosis has a high mortality rate of 76% and the lungs are the second most commonly reported site after the sinuses.^[5]^ The clinical features of pulmonary mucormycosis are nonspecific and cannot be distinguished from pulmonary aspergillosis. High-grade fever and a nonproductive cough are the most common symptoms.^[5]^

Most of the signs of pulmonary mucormycosis on CT scan are indistinguishable from other invasive pulmonary fungal infections, with the presence of infiltrates, consolidation, nodules, cavitations, atelectasis, effusion, posterior tracheal band thickening and hilar or mediastinal lymphadenopathies. Only the reversed halo sign, a focal round area of ground-glass attenuation surrounded by a ring of consolidation, appears more specific and is highly suggestive of pulmonary mucormycosis.^[13]^

Evidence of mucormycosis on cultures or on histopathological examination is required for a definitive diagnosis.^[14]^ PCR detection of Mucorales DNA in blood samples is now recognized as the earliest biological indication of mucormycoses^[15–17]^ and could even precede the diagnosis of proven mucormycosis by an average of 9 days.^[16]^

Mucormycosis tends to occur late in autologous stem cells recipients for various diseases (median interval, 412 days; range, 190–2254 days).^[18]^ In this case, the patient presented with acute respiratory distress only 6 days after PBSC reinjection, which underscores the crucial role of prior immunosuppression, especially prolonged use of corticosteroids, which was the case for our patient.

Management guidelines suggest amphotericin B as the first-line treatment (daily dose 5 mg/kg) combined with a surgical procedure, when necessary, and then a switch to posaconazole as a maintenance treatment.^[19]^ Even so, mortality rates are still high (61%).^[20]^

Early antifungal therapy is essential, as soon as the diagnosis is suspected, without waiting for biological evidence. In fact, Chamilos et al^[21]^ showed that delaying the administration of amphotericin B–based regimens by 5 days is associated with a 2-fold increase in mortality.

In hematological patients, the risk of mucormycosis after stem cell transplantation is well-documented, with a postallograft incidence estimated at 0.3%,^[3]^ which represents approximately 8% of the postallograft invasive fungal infections.^[18]^ Less data is available on the occurrence of mucormycosis after AHSCT, but the incidence appears to be lower with 7.8% of the post-autograft invasive fungal infections.^[4]^ In systemic autoimmune diseases, the onset of mucormycosis remains uncommon and appears to be related to the use of corticosteroids.^[5,22]^ In a series of 24 cases of autoimmune disease patients with mucormycosis,^[23]^ 83% had systemic lupus erythematosus. All the subjects had been exposed to corticosteroids and 6 had received additional CPM. In autoimmune diseases, mucormycosis symptoms can mimic a flare of the underlying disease.

In France, between 1996 and 2018, 75 AHSCT for severe SSc were performed^[11]^ with no report of increased risk for post-transplantation invasive fungal infection. In the various other trials, which showed the benefit of AHSCT in SSc patients,^[6–8]^ a higher proportion of viral infections were described but no increased risk for fungal infection has been noted.

## 4 Conclusion

In light of the increase in the availability of AHSCT for severe SSc clinicians should be aware of this rare post-transplant fungal infection and that early initiation of adequate therapy for patients with clinical and radiological features consistent with mucormycosis could change the outcome.

## Author contributions

**Conceptualization:** Grégory Pugnet.

**Supervision:** Grégory Pugnet.

**Validation:** Grégory Pugnet.

**Writing – original draft:** Xavier Boumaza.

**Writing – review & editing:** Xavier Boumaza, Lucie Lelievre, Sarah Guenounou, Cécile Borel, Anne Huynh, Guillaume Beziat, Karen Delavigne, Damien Guinault, Marie Garric, Marie Piel-Julian, Kim Paricaud, Guillaume Moulis, Leonardo Astudillo, Laurent Sailler, Dominique Farge, Grégory Pugnet.
